# Sulfadiazine resistance in *Toxoplasma gondii*: no involvement of overexpression or polymorphisms in genes of therapeutic targets and ABC transporters

**DOI:** 10.1051/parasite/2013020

**Published:** 2013-05-27

**Authors:** Christelle Doliwa, Sandie Escotte-Binet, Dominique Aubert, Virginie Sauvage, Frédéric Velard, Aline Schmid, Isabelle Villena

**Affiliations:** 1 Laboratoire de Parasitologie-Mycologie, EA 3800, SFR CAP-Santé FED 4231, UFR Médecine, Université de Reims Champagne-Ardenne 51 rue Cognacq-Jay 51095 Reims Cedex France; 2 CRB Toxoplasma et CNR de la Toxoplasmose, Hôpital Maison Blanche 45 rue Cognacq- Jay 51092 Reims Cedex France; 3 Plateforme d’Imagerie Cellulaire et Tissulaire 51 rue Cognacq-Jay 51096 Reims Cedex France; 4 EA 4691 BIOS : Biomatériaux et inflammation en site osseux, SFR CAP-Santé FED 4231, Université de Reims Champagne-Ardenne 51 rue Cognacq-Jay 51095 Reims Cedex France

**Keywords:** *Toxoplasma gondii*, sulfadiazine resistance, *dhps*, *dhfr*, ABC transporters

## Abstract

Several treatment failures have been reported for the treatment of toxoplasmic encephalitis, chorioretinitis, and congenital toxoplasmosis. Recently we found three *Toxoplasma gondii* strains naturally resistant to sulfadiazine and we developed *in vitro* two sulfadiazine resistant strains, RH-R^SDZ^ and ME-49-R^SDZ^, by gradual pressure. In *Plasmodium*, common mechanisms of drug resistance involve, among others, mutations and/or amplification within genes encoding the therapeutic targets *dhps* and *dhfr* and/or the ABC transporter genes family. To identify genotypic and/or phenotypic markers of resistance in *T. gondii*, we sequenced and analyzed the expression levels of therapeutic targets *dhps* and *dhfr*, three ABC genes, two Pgp, *TgABC.B1* and *TgABC.B2*, and one MRP, *TgABC.C1*, on sensitive strains compared to sulfadiazine resistant strains. Neither polymorphism nor overexpression was identified. Contrary to *Plasmodium*, in which mutations and/or overexpression within gene targets and ABC transporters are involved in antimalarial resistance, *T. gondii* sulfadiazine resistance is not related to these toxoplasmic genes studied.

## Abstract (fr)

De nombreux échecs thérapeutiques ont été rapportés dans le traitement de la toxoplasmose cérébrale, des choriorétinites et de la toxoplasmose congénitale. Récemment, nous avons identifié trois souches de *Toxoplasma gondii* naturellement résistantes à la sulfadiazine et nous avons développé *in vitro*, par pression médicamenteuse croissante, deux souches résistantes à la sulfadiazine, RH-R^SDZ^ et ME-49-R^SDZ^. Chez *Plasmodium*, les mécanismes de résistance impliquent, entre autres, la mutation et/ou l’amplification de gènes codant pour les cibles thérapeutiques, *dhps* et *dhfr*, et/ou les gènes de la famille des ABC transporteurs. Afin d’identifier des marqueurs génotypiques et/ou phénotypiques de résistance de *T. gondii*, nous avons séquencé et analysé les niveaux d’expression des gènes cibles, *dhps*, *dhfr*, et de trois gènes de transporteurs ABC, deux Pgp, *TgABC.B1* et *TgABC.B2*, et une MRP, *TgABC.C1*, par comparaison des souches sensibles et résistantes à la sulfadiazine. Aucun polymorphisme ni surexpression de gènes n’ont été identifiés. Contrairement à *Plasmodium*, dont les mutations et/ou surexpressions des gènes cibles et ABC transporteurs sont impliquées dans la résistance aux antipaludiques, les mécanismes de résistance à la sulfadiazine chez *T. gondii* ne sont pas liés à ces gènes toxoplasmiques étudiés.

## Introduction

The apicomplexan *Toxoplasma gondii*, an obligate intracellular parasite, can infect humans and a wide range of vertebrates leading to toxoplasmosis. This generally benign affection can cause severe life-threatening disease, particularly in immunocompromised patients and in congenitally affected children [17]. The population structure of *T. gondii* consists of three main clonal lineages (Type I (including RH, a highly virulent strain), Type II (including avirulent strains like ME-49 and PRU), and Type III (including avirulent strains like NED)) correlated with virulence expression in mice [5]. Recently, a study revealed a biphasic pattern consisting of regions in the Northern Hemisphere where a few highly clonal and abundant lineages predominate; elsewhere, and especially in portions of South America, they are characterized by a diverse assemblage of less common genotypes that show greater evidence of recombination [14].

Treatment of toxoplasmosis usually uses a combination of a sulfamide with pyrimethamine, which has a remarkable synergistic activity against the replicating form of *T. gondii*, through the sequential inhibition of parasite dihydropteroate synthase (DHPS) and dihydrofolate reductase (DHFR). These two major enzymes are responsible for the synthesis of the folate compounds that are essential for parasite survival and replication. However, several treatment failures have been reported for treatment of toxoplasmic encephalitis, chorioretinitis and congenital toxoplasmosis [16]. Whether these failures are related to host factors (drug intolerance, malabsorption, poor compliance) and/or to the development of drug-resistant parasites or a lower susceptibility of the parasite strain is debated. Recently, *in vitro* susceptibilities of 17 *T. gondii* strains belonging to various genotypes were evaluated with the widely used anti-toxoplasmic drugs including sulfadiazine, pyrimethamine, and atovaquone [7]. Some variability in the susceptibilities of *T. gondii* strains to pyrimethamine and atovaquone were found but with no clear evidence of drug resistance. On the other hand, higher variability was found for sulfadiazine with *in vitro* resistance for three strains, TgH 32006, previously described as RMS-1995-ABE, TgH 32045, previously described as RMS-2001-MAU, and TgA 103001, previously described as B1, not correlated to strain genotypes or growth kinetics [7]. Moreover, in order to understand sulfadiazine resistance mechanisms in *T. gondii*, we developed *in vitro* two sulfadiazine-resistant strains, named RH-R^SDZ^ and ME-49-R^SDZ^, by drug pressure [3].

The molecular basis of resistance to antifolates is well documented in *P. falciparum* and consists of point mutations in genes encoding for both *dhps* and *dhfr*. Resistance to pyrimethamine has been shown to result from a mutation in the DHFR enzyme, changing Ser108 to Asn108, and subsequent mutations (N51I, C59R, I164L, and A16V) can greatly increase the level of resistance to this drug. Resistance to sulfonamides and sulfones has been demonstrated to result from mutations within DHPS, such as amino acid changes at five positions (S436A/F, A437G, K540E, A581G, A613/T) [2]. In *T. gondii*, Aspinall *et al*. (2002) [2] demonstrated by direct sequencing of PCR products the presence of six mutations at positions 407, 474, 560, 580, 597, and 627 within DHPS. Only the mutation at position 407, which is equivalent to the 437 position in *Plasmodium*, was reported as being associated with sulfonamides resistance. This mutation was also retrieved in the laboratory-induced sulfamethoxazole-resistant strain (R-Sul^R^-5) [8].

We previously demonstrated that accumulation and efflux of xenobiotics from parasites were modulated by P-glycoprotein (Pgp) and Multidrug resistance-associated protein (MRP) inhibitors, indicating their presence and activity in *T. gondii* [10]. Pgp and MRP proteins belong to the ATP-binding cassette (ABC) superfamily of transporters. So far, we have identified in the *T. gondii* genome 24 genes related to the ABC whose expression was detected both in tachyzoite and bradyzoite infectious stages for the three genotypes (I, II, and III) [12]. Among these 24 genes, two encode for whole Pgps: *TgABC.B1* (1345 amino acids) [10] and *TgABC.B2* (1407 amino acids) and one encodes for a MRP, *TgABC.C1* (1883 amino acids). Pgp and MRP are widely reported to export xenobiotics and cause drug resistance in tumor cells [1] and protozoan parasites [11] and lead to drug resistance by increasing drug efflux from the cell, thus lowering the effective intracellular drug concentration. The increased activities of the ABC transporters could be due to an increased amount of proteins due to gene amplification or overexpression associated or not associated with point mutations in the genomic sequence. In *P. falciparum*, antimalarial resistance involves mutations and/or amplification of one Pgp and MRP genes, *PfABCB1* (alias Pgh1 and *PfMDR1*) and *PfABCC1* (alias *PfMRP*), respectively. Mutations in *PfABCB1* are identified in clinical isolates from different geographical areas. Polymorphisms are observed at five positions – codons 86, 184, 1034, 1042, and 1246. *PfABCB1* overexpression is the only mechanism suggested to date involved in mefloquine-resistant parasites [9]. Concerning *PfABCC1*, mutations at positions 191His and 437Ser are found to be linked 100% to decreased quinolone resistance in southeastern Iranian isolates [15].

In our present study, we sequenced and analyzed the expression levels of the therapeutic targets *dhps* and *dhfr* and three ABC transporters, *TgABC.B1*, *TgABC.B2* and *TgABC.C1*, in sulfadiazine-sensitive and resistant *T. gondii* strains to identify genotypic and/or phenotypic markers of resistance.

## Material and methods

### Cell culture

*T. gondii* tachyzoites were maintained on Vero cell monolayers (ATCC, CCL-81) at 37 °C in a 5% CO_2_ humidified incubator. Cells and parasites were grown in complete medium: Iscove’s Modified Dulbecco’s Medium/Glutamax (IMDM; Invitrogen, France) supplemented with 2% (v/v) fetal calf serum (Biowest, France) and antibiotics (100 IU/mL penicillin and 0.1 mg/mL streptomycin) (GIBCO) as previously described [3].

### Polymorphisms analysis

Identification of polymorphic sites of *dhps*, *dhfr*, *TgABC.B1*, *TgABC.B2*, and *TgABC.C1* genes was carried out by using PCR amplification and direct sequencing [13]. Strain polymorphisms were analyzed by alignment of the nucleotide sequences according to the ClustalW multiple sequence alignment program at the website of EMBL-EBI (http://www.ebi.ac.uk//clustalw/index.html).

### qRT-PCR analysis

The protocol used was previously described [13]. PCR primers (Invitrogen™ Life Technologies, France) were designed using Primer express 2.0 (Applied Biosystems, USA) to specifically amplify sequences of *dhps*: 5′-TCA TTT CCG TTG ACA CCA TGA-3′ (forward) and 5′-TCT CCG GTC TGG TCG TTC AC-3′ (reverse), *dhfr*: 5′-CTG GAG GAA GAG TAC AAG GAT TCT GA-3′ (forward) and 5′-AAG CAA CGC CCA GAG ACA-3′ (reverse), *TgABC.B1*: 5′-GCG TGT GTT TGC ACT GAT TGA-3′ (forward) and 5′-TTG CGT TGT CGC TGA ACT TC-3′ (reverse), *TgABC.B2* : 5′-CGA TCG TGC AGA TGC TTC AA-3′(forward) and 5′-GCT GTG CAC GCA GAT ACT GAA T-3′ (reverse), *TgABC.C1*: 5′-ACA CTC TCC CTT CAT TCA CAA G-3′ (forward) and 5′-CAG AAG GTG AAT CAC TGG AAT GG-3′ (reverse), and the *toxoplasma β-tubulin*: 5′-TCT TCC GCC CTG ACA ACT TC-3′ (forward) and 5′-CCG CAC CCT CAG TGT AGT GA-3′ (reverse). Results are representative of at least five independent experiments and presented as median ± interquartile spaces (IQs). *p < 0.05 represent significant difference between strains (Non-parametric exact Wilcoxon-Mann-Whitney test).

### Nucleotide sequence data

Nucleotide sequence data reported in this paper are available in the GenBank™, EMBL, and DDJB databases under the accession numbers: EU213065, EF418617, FJ201251, EU213066, EF418618, EJ201252, EU213067, EF418619, FJ201253, GQ415579, GQ397454, FJ201257, FJ215662, GQ865628, GQ415585, GQ397458, FJ201258, GQ865630, GQ865629, GQ415580, GQ397459, FJ201255, FJ201256, FJ201254, GQ415574, GQ395774.

## Results and discussion

**Figure 1.**
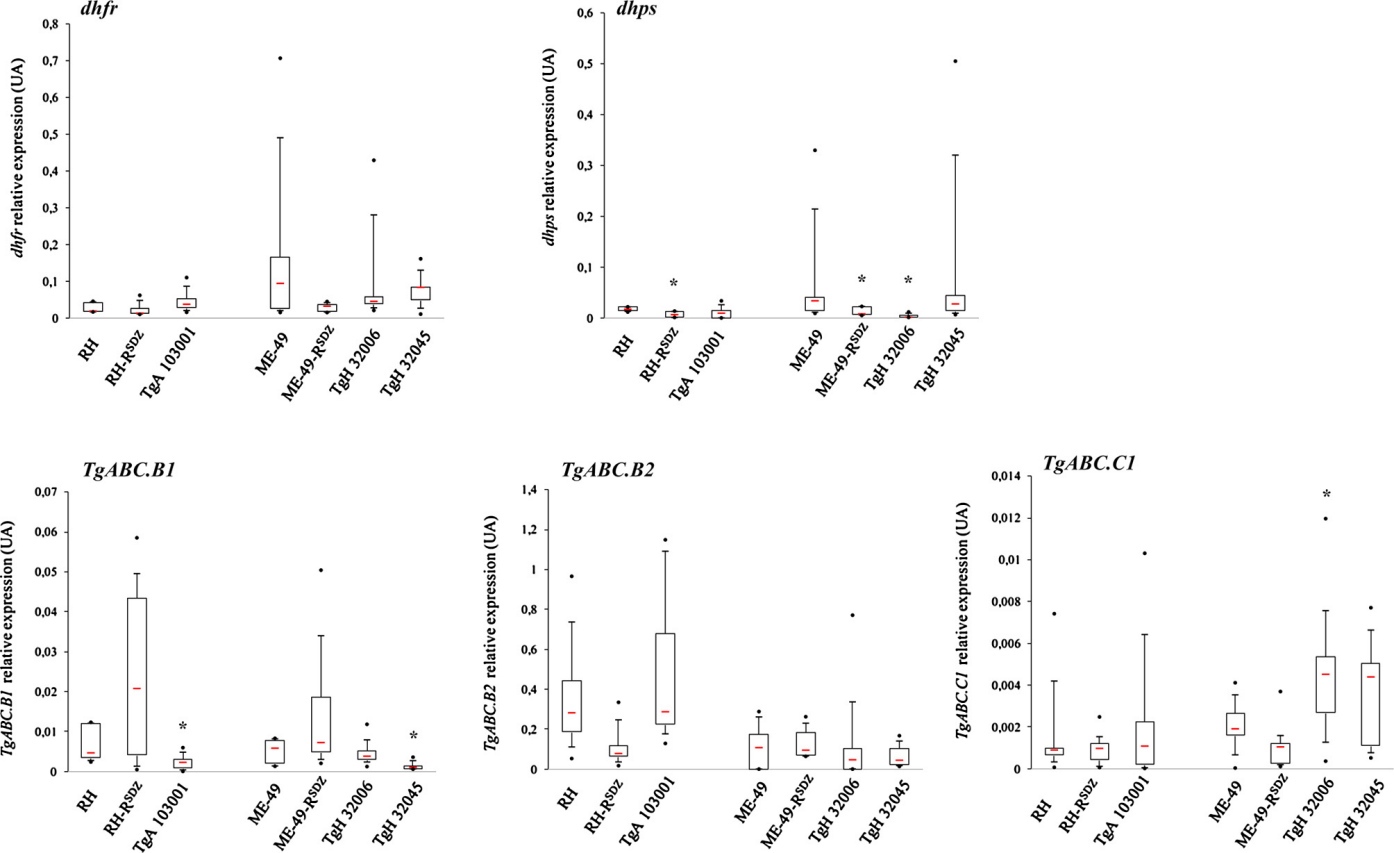
Relative expression of *dhps*, *dhfr*, *TgABC.B1*, *TgABC.B2*, and *TgABC.C1* genes in two sensitive strains H (I) ME-49 (II), and the induced-resistant strains, RH-R^SDZ^ and ME-49-R^SDZ^, and naturally resistant strains TgA 103001, TgH 32006, and TgH 32045 by qRT-PCR analysis. Red bars represent median value. Black points represent maximum and minimum values. Black bars represent first and tenth decile and limits of white rectangle represents first and third quartile.

To identify genotypic and/or phenotypic markers of resistance, we sequenced and analyzed the expression levels of the therapeutic targets *dhps* and *dhfr* on sensitive strains representative of the three major genotypes (Type I (RH), Type II (ME-49 or PRU), and Type III (NED)), compared to the three naturally resistant strains described (TgA 103001 (Type I), TgH 32006 (Type II), and TgH 32045 (Type II variant)). For the polymorphisms analysis, the Type II strain ME-49 was considered as reference; genotype II strains were found in 95% of cases of toxoplasmosis in France. The complete sequence of the 6 exons of the *dhps* gene showed three identical mutations in the exons 2 (E474D), 4 (R560K), and 5 (A597E, two silent mutations) of the sensitive strain RH as well as in the resistant strain TgA 103001, one of the three naturally resistant strains to sulfadiazine (Table 1). This mutation was also found in one recombinant Type I/III strain (TgH 32005A, previously described as RMS-1994-LEF) and in one atypical strain isolated in French Guyana (TgH 18007A, previously described as GUY-2003-MEL), both of them tested as sensitive to sulfadiazine [7]. In the resistant strain TgH 32006, one mutation converting Alanine to Valine at position 587 was found in exon 5 [7]. The significance of this new mutation on the *dhps* gene demonstrated in one of the three resistant strains remains to be determined. In addition, no mutation was found at position 407 in the three resistant strains analyzed. As previously described [7], one silent mutation in exon 3 (156L) of the *dhfr* gene was found in the two Type I strains, the sensitive strain RH and the resistant strain TgA 103001. ABC transporters have been reported to be involved in drug resistance in protozoa [11]. We have sequenced and analyzed the expression levels of *TgABC.B1*, *TgABC.B2*, and *TgABC.C1* on three sensitive and three naturally resistant strains. The sequencing of *TgABC.B1* (35 exons), *TgABC.B2* (33 exons) and *TgABC.C1* (9 exons) coding regions on the three major genotypes – Type I (RH), Type II (PRU), and Type III (NED) – shows 26, 29, and 27 single nucleotide polymorphisms, respectively*. TgABC.B1* shows silent mutations at 24 sites, discriminating the RH, PRU, and NED strains. Two mutations, in the exons 1 (A9T) and 35 (K1324Q), lead to changes in amino acids which helped distinguish between Type II and non-Type II *T. gondii* strains (Table 1). Several silent mutations were found in the *TgABC.B1* gene according to different strain genotypes. Concerning *TgABC.B2*, 22 silent mutations sites, of which seven single nucleotide polymorphisms that help distinguish between Type I and non-Type I *T. gondii* strains, were identified. TgH 32045 presented one mutation in exon 18 (L729M) found in the Type I strains. The *TgABC.C1* gene shows 17 silent mutations in the coding region, of which 10 mutation sites lead to changes in amino acids, discriminating the Type I and non-Type I strains (Table 1). TgH 32045 presented one mutation in exon 9 (H1659Q) found in the Type I sulfadiazine-resistant strain (TgA 103001). This mutation was retrieved in all Type I strains subsequently studied (except RH), as well as on atypical strains from special geographical regions, like French Guyana and Brazil (*data not shown*). The low polymorphism percentage observed for the different genes studied is in concordance with the genetic variation level estimated to be less than 2% among the predominant clonal lineages [4]. The expression level of each therapeutic target was analyzed using standard semi-quantitative real-time RT-PCR for all the strains studied. After normalizing transcript levels of *dhps* and *dhfr* to *β-tubulin*, no significant variation of *dhfr* gene expression was observed between resistant and sensitive strains (Figure 1). However, we observed a significant decrease (*p* < 0.05) of *dhps* gene expression in the resistant strain RH-R^SDZ^ in comparison to the sensitive RH strain and in the two Type II resistant strains TgH 32006 and ME-49-R^SDZ^ in comparison to the sensitive ME-49 strain. These results were not consistent with overexpression of therapeutic targets found in *Plasmodium*. Hence, no polymorphism or overexpression of therapeutic targets is involved in *T. gondii* sulfadiazine resistance. The RNA expression levels from the two Pgp and the MRP demonstrate that gene expression seems correlated with the strain genotype, as observed with Type I strains, which present the highest level of expression for the *TgABC.B2* gene. The virulent strains are characterized by a high growth rate compared to avirulent strains, which could involve a greater metabolism and therefore an efficient detoxification mechanism. This could explain the higher expression of *TgABC.B2* in the sensitive RH strain and the resistant TgA 103001 strain (Figure 1). As gene overexpression, including some ABC genes (ABC.G5, ABC1, ABC2), especially for RH versus other Type I isolates, has been previously described [6], we analyzed the *TgABC.B2* gene on ENT strain (Type I). No variation of *TgABC.B2* gene expression was observed; RH and ENT strains have the same *TgABC.B2* gene expression variability (*data not shown*). Moreover, we observed a statistical decrease (*p* < 0.05) in *TgABC.B1* gene expression for the resistant strains TgA 103001 (Type I) and TgH 32045 (Type II variant) compared to the sensitive strains RH (Type I) and ME-49 (Type II). Interestingly, we observed a significant overexpression of *TgABC.C1* (*p* < 0.05) in the resistant strain TgH 32006 compared to the sensitive strain ME-49, but no significant variation of this gene was observed in the other two naturally resistant strains, TgA 103001 and TgH 32045. Moreover, no significant overexpression of *TgABC.B1* and *TgABC.C1* was observed in the two resistant-induced strains, RH-R^SDZ^ and ME-49-R^SDZ^ (Figure 1).

**Table 1.** Polymorphisms in the therapeutic targets DHPS and DHFR and the three ABC proteins, TgABC.B1, TgABC.B2, and TgABC.C1, for three sensitive and naturally resistant strains representative of the three major genotypes (I, II, and III) in *T. gondii*. Only the polymorphisms leading to amino acid changes are represented.

	Genotype	Sulfadiazine susceptibility	*TgABC.B1*	*TgABC.B2*	*TgABC.C1*	*dhps*	*dhfr*
RH	I	Sensitive	* (20) T9A, K1285Q EU213065.1	* (22) S2N, S267N, G368D, I509T, L729M, M848L, L883I EF418617.1	* (19) K7E, D491E, T665S G672E, H954P, G968A E1125A, S1718R V1722A, P1785S FJ201251.1	*(2) E474K, R560K A597E	*(1)
PRU	II	Sensitive	0	0	0	0	0
			EU213066.1	EF418618.1	FJ201252.1	GQ415579.1	GQ397454.1
NED	III	Sensitive	* (13)	0	0	0	0
			T9A, K1285Q EU213067.1	EF418619.1	FJ201253.1	GQ415579.1	GQ397454.1
TgA 103001	I	Resistant	* (19) T9A, K1285Q FJ201255.1	* (23) S2N, S267N, G368D, I509T, G816E, M848L, L883I FJ201256.1	* (23) K7E, D491E, G782A H954P, G968A E1125A, G1440A, H1659Q, S1718R, V1722A, P1785S FJ201254.1	* (2) E474K, R560K, A597E GQ415574.1	* (1) GQ415574.1
TgH 32006	II	Resistant	0	0	0	A587V	0
			FJ201257.1	FJ215662.1	GQ865628.1	GQ865628.1	GQ397458.1
TgH 32045	II Variant	Resistant	* (1)	*(1) L729M	K642E, H1659Q	0	0
			FJ201258.1	GQ865630.1	GQ865629.1	GQ415580.1	GQ397459.1

* corresponds to silent mutations. GenBank accession numbers are indicated for each case.

In conclusion, we demonstrated that, in the case of *T. gondii*, sulfadiazine resistance does not involve polymorphisms and/or overexpression in *dhfr*, *dhps*, *TgABC.B1*, and *TgABC.B2* genes contrary to *P. falciparum*. These results imply that resistant mechanisms in *T. gondii* are different. Interestingly, an overexpression of *TgABC.C1* was observed in the Type II resistant strain TgH 32006, further studies are needed to clarify its involvement in resistance mechanisms. Studies are underway to investigate the drug resistance mechanisms in *T. gondii* using a microarray approach by comparison between sensitive and sulfadiazine-resistant strains. The identification of genes associated with sulfadiazine resistance will allow us to understand the resistance mechanisms implicated.
